# Sensory appendage protein triggers alarm to pyrethroid in Indian malarial vector *Anopheles culicifacies*

**DOI:** 10.1371/journal.pone.0333483

**Published:** 2025-10-07

**Authors:** Vaishali Saini, Pooja Rohilla, Vartika Srivastava, Gitanjali Tandon, Pooja Yadav, Nirmala Sankhala, Tanvi Singh, Gunjan Sharma, Suchi Tyagi, Tanwee Das De, Rajnikant Dixit

**Affiliations:** 1 Laboratory of Host-Parasite Interaction Studies, Department of Vector Genomics, ICMR-National Institute of Malaria Research, Dwarka, New Delhi, India; 2 Academy of Scientific and Innovative Research (AcSIR), Ghaziabad, Uttar Pradesh, India; 3 Department of Medical Entomology and Zoology, ICMR-National Institute of Virology, Microbial Containment Complex, Pune, Maharashtra, India; Beni Suef University Faculty of Veterinary Medicine, EGYPT

## Abstract

Sensory appendage proteins (SAPs), members of the odorant-binding protein family, mediate chemical communication that is vital for mosquito survival and vector competence. Among the six identified SAP members, *AcSAP1* and *AcSAP2* exhibited dominant expression in olfactory and leg tissues of *Anopheles culicifacies*. Our findings reveal that the expression of *AcSAP1*, and to a lesser extent *AcSAP2*, is significantly modulated by the circadian rhythm, with peak expression coinciding with the nocturnal host-seeking behavior of female mosquitoes. A delayed host attraction response by the *AcSAP1*-silenced female mosquitoes accounts for *AcSAP1’s* potential role in host-seeking activities. *In silico* prediction data showed strong binding affinity to synthetic pyrethroid (deltamethrin); therefore, we tested the molecular responses of SAP to insecticide exposure. Strikingly, our WHO tube test bioassay-based experimental evaluation of mosquitoes’ survival and expression modulation in the olfactory, wings, and legs highlighted SAPs’ ability to sense toxic chemical cues. Finally, coupled with confocal microscopy analysis, our notable observation of a significant increase in *AcSAP1* expression in the eyes of mosquitoes challenged with deltamethrin suggests a potential role in integrating visual and chemosensory cues. Our results highlight the multifaceted functions of SAPs, demonstrating their dual roles in host-seeking and insecticide avoidance, which have significant implications for understanding mosquito biology and developing novel vector control strategies.

## Introduction

To feed, adapt, and survive under different environmental conditions, mosquitoes have developed an efficient and sophisticated sensory system. They rely on their sense of smell to navigate their surroundings and find appropriate food sources, identify mates, and ovipositing sites. The primary source of nourishment and energy for both male and female mosquitoes is nectar sugar [1]. However, female mosquitoes consume blood to reproduce and maintain the gonotrophic cycle. During the blood-feeding states, a mosquito’s host-seeking behavior is dominantly guided by the controlled action of the neuro-olfactory system [2], which integrates external chemical cues with internal physiological states. Since host-seeking behaviour is central to malaria transmission and mosquitoes rely on olfactory components to locate the human host, unravelling the molecular basis of mosquito olfaction may provide a new strategy to disrupt the host-seeking behaviour and subsequently reduce malaria transmission.

The mosquito olfactory system comprises three primary peripheral sensory organs—the antennae, maxillary palps, and proboscis that collectively detect and process host-associated cues [3]. Within these structures, chemoreception is initiated when volatile odor molecules from the external environment are captured and transported through the sensillar lymph, enabling subsequent detection and signal transduction. This initial step is largely mediated by two major classes of small soluble proteins, odorant-binding proteins (OBPs) and chemosensory proteins (CSPs). These proteins, abundant in sensillar lymph, capture, solubilize, and transport odorant and pheromones to membrane-bound receptors located on the dendrites of olfactory sensory neurons. By facilitating the ligand–receptor interactions, OBPs and CSPs play a pivotal role in shaping mosquito host-seeking and blood-feeding behavior [4]. Subsequent odor recognition and signal transduction are mediated by distinct receptor families, including odorant receptors (ORs), ionotropic receptors (IRs), and sensory neuron membrane proteins (SNMPs), which decode diverse chemical stimuli and activate olfactory signalling pathways [5].

Among these two classes of small soluble proteins, OBPs have been extensively studied. OBPs are relatively larger (~14–18 kDa), and are characterized by six conserved cysteine residues forming three to four disulfide bonds, which confer structural stability and make them ideal for transporting odorants in volatile environments. In contrast, CSPs are smaller in size (~10–12 kDa), and carry four cysteines and contain a characteristic OS-D (Olfactory Segment D) domain (4). This OS-D domain is a conserved structural motif found in a subset of insect chemosensory proteins (CSPs), including those often referred to as Sensory Appendage Proteins (SAPs). While insect studies suggest that CSPs exhibit a broader tissue expression and may participate in diverse biological processes such as development and detoxification [6] their precise functional contributions to mosquito chemoreception and behavior remain insufficiently defined.

Genomic studies in *Anopheles* mosquitoes have identified at least six chemosensory protein (CSP) transcripts (CSP1–CSP6), most of which are predominantly expressed in the olfactory system [7]. A comparative genomic analysis across 22 mosquito species *viz*. Aedes, Culex, and Anopheles revealed that these CSPs are highly conserved across *Anopheles*, with strong evidence of purifying selection acting on them]. Our RNA-Seq analysis of *An. culicifacies* revealed a similar repertoire, with two members of CSPs i.e. *AcSAP-1* (*ACUA018714*) and *AcSAP-2* (*ACUA013409*), showing high expression in both the legs and olfactory tissues. Interestingly, their rhythmic fluctuation during the late-night hours suggests a potential role in regulating host-seeking behavior [8]. The detection of SAP expression in the legs further indicates that their function may extend beyond classical olfaction. Supporting this broader perspective, recent work in *An. gambiae* have shown that SAP proteins contribute not only to chemoreception but also to insecticide resistance, although the underlying mechanism remains poorly defined [9].

*An. culicifacies* is responsible for transmitting approximately 65% of malaria cases in rural India [10]. However, the growing concern of resistance to pyrethroid-based long-lasting insecticidal nets (LLINs) and indoor residual spraying (IRS), together with limited understanding of the behaviour and physiological mechanisms involved in malaria transmission, poses a major challenge for effective control of this species. Although studies in *An. gambiae* have reported the expression of sensory appendage proteins (SAPs) in the legs and implicated them in insecticide resistance [9], their functional relevance in *An. culicifacies* remain largely unexplored. In this study, we investigated the role of SAPs in *An. culicifacies* through transcriptional profiling and functional assays. Our results demonstrate that SAPs are not only associated with host-seeking behavior but also contribute to rapid hypersensitization of peripheral sensillum organs in response to pyrethroid exposure. These findings suggest that SAPs may act as early-warning detectors against toxicants, offering new insights for understanding resistance mechanisms and guiding the development of strategies to mitigate the spread of pyrethroid resistance in natural mosquito populations.

## Materials and methods

### Mosquito rearing and maintenance

#### An.

*culicifacies* (sibling species A) was reared and maintained in the central insectary of the National Institute of Malaria Research under standard rearing conditions of 28 ± 2°C, relative humidity 60–80%, and 12:12 hr light/dark cycle. The aquatic stages were reared in enamel trays using water supplemented with a mixture of dog food and fish food. After emergence, adults were kept in mosquito cages and fed on cotton swabs dipped in a 10% sugar solution. All protocols for mosquito rearing and cyclic colony maintenance were duly approved by the institute’s ethical committee [NIMR/IAEC/2017–1/07] [11].

### Tissue collection and RNA extraction

Various tissues, including the complete olfactory system (antennae, maxillary palp, proboscis, and labium), brain, and legs, were dissected from 3-4-day-old adult female mosquitoes anesthetized on ice. These tissues were immediately collected in Trizol Reagent (RNAiso Plus, Takara Bio, Kusatsu, Japan) for RNA extraction. In addition, head tissues containing the olfactory appendages were dissected at different ages (1–9 days old) and at various Zeitgeber time (ZT) points to capture circadian variations. The 24 h time scale of the light-dark (LD) cycle is represented as different Zeitgeber time (ZT), where ZT0 indicates the end of dawn transition, ZT11 is defined as the start of the dusk transition, and ZT12 is defined as the time of lights off [3]. Developmental stages of *An. culicifacies viz.* egg, larvae (stages I − IV), and pupae were independently collected in Trizol after carefully removing excess water using filter paper. Total RNA was isolated by the standard Trizol method as described previously [12] and quantified by using a Nanodrop 2000 (ND-2000c) spectrophotometer (Thermo Scientific, USA).

### cDNA preparation and gene expression analysis

Approximately 1 μg of total RNA was used to synthesize the first strand of cDNA using the Primescript™ 1st strand cDNA Synthesis Kit (Takara Bio, Kusatsu, Japan) following the manufacturer’s protocol. Routine differential gene expression analysis was performed by RT-PCR. Relative gene expression analysis was carried out using SYBR Green qPCR Master Mix (Takara Bio Inc., Kusatsu, Japan) on a Bio-Rad CFX96™ Real-Time system (USA). The PCR cycling conditions included an initial denaturation at 95°C for 5 min, followed by 40 cycles of 95°C for 10s, 52°C for 15s, and 72°C for 22s. Fluorescence readings were collected at 72°C in each cycle. A final dissociation step (95°C for 15s, 55°C for 15s, and 95°C for 15s) was performed to generate the melting curve and confirm amplification specificity [13]. Reproducibility was ensured by performing three independent biological replicates for each experimental group. Actin, which showed stable expression among candidate reference genes (RpS7 and CYP450), was used as the internal control. Relative expression was calculated using the 2–ΔΔCt method [14], and expression graphs were plotted in GraphPad Prism 10 software.

### dsRNA-mediated gene silencing

To test the effect of *AcSAP1*-knockdown (ACUA018714), an *in vitro* transcription reaction was performed using the Transcript Aid T7 high-yield transcription kit (Cat# K044, Ambion, USA). Approximately 69nl of purified dsRNA at a concentration of ~3 μg/μl for the target gene was injected into the thorax of cold anesthetized 1–2-day(s)-old female mosquitoes using a Nano-injector (Drummond Scientific, CA, USA). For the control group, age-matched mosquitoes were injected with bacterial-origin *LacZ* dsRNA. Three days post dsRNA injection, both control and experimental groups of mosquitoes were used for phenotypic assays. Simultaneously, to examine the silencing efficiency, olfactory and leg tissues were collected in Trizol from 20 mosquitoes each from the control and SAP-silenced mosquitoes for RNA extraction and cDNA preparation. The efficiency of silencing was evaluated using quantitative real-time PCR as described above. Out of four independent experimental assays, three successful experiments were included for data analysis.

### Host-seeking behavioral assay

To assess the behavioural and phenotypic effect, a host-proximity assay was carried out to compare the host-seeking efficiency between control and *AcSAP1-silenced* mosquito groups. Human host proximity assay was performed by minor modification of previously described methods [15]. The two-port mosquito behavioural assay chamber was designed with dimensions of 30 cm in height, 40 cm in width, and 30 cm in length. It was fabricated using flexible glass material. The chamber includes a central splitting door, creating two equal-sized compartments, each with dimensions of 30 cm in height, 20 cm in width, and 30 cm in length. One compartment was designated for the control mosquitoes, while the other was for *AcSAP1*-silenced mosquitoes. Each chamber is built with an arm (L × D: 27 × 12 cm) for placing human hands.

Before the commencement of the behavioural experiment, 25 non-blood-fed (3−4 days old) female mosquitoes from both the control and the *AcSAP1*-silenced group were released into their respective chambers and allowed to acclimatize for 5−10 minutes. After acclimatization, the hands of the volunteer were placed into the arms of the behavioural assay chamber. The usage of soap or any cosmetic products by the volunteers participating in this assay was restricted for at least 24 hours. To avoid bias, the assay setup was kept blinded in a way that the person (observer), recording the count of mosquito attraction does not know the nature of the experimental mosquito group, i.e., control or silenced [16] A gauze barrier was incorporated into the arm (at 15 cm), and the distance between the hand and the barrier was 0.5 cm to avoid direct landing and biting by mosquitoes on the hand. The protocol was duly approved by the Institute Ethics Committee (PHB/NIMR/EC/2021/150), and the Institute Biosafety Committee (IBSC/NIMR/2022/06), and the study was conducted from 01/11/2022–31/10/2023. Considering the nocturnal behavioural activity, the assay was conducted at ZT16−18, and data were recorded manually for 8−10 minutes. The number of mosquitoes that responded and were attracted to the hands was counted manually. At an interval of every 30sec-1 min, we counted the number of mosquitoes in real-time that were attracted to the volunteer’s hands, landed on the gauze, and attempted to bite. The difference in host-seeking efficiency was calculated as the percentage of attracted mosquitoes by quantifying the ratio of landed and total number of mosquitoes used in the respective assay. The experiment was repeated three times (three cohorts of mosquitoes).

### In-silico prediction of SAP binding affinity with deltamethrin

The protein sequences of SAP1, SAP2, and SAP3 were subjected to physiochemical analysis with the help of ProtParam tool (https://web.expasy.org/protparam/) [17] at EXPASY while the Cellular localization of SAP proteins was predicted by using web servers CELLO v.2.5 (http://cello.life.nctu.edu.tw/) [18] and WoLF PSORT (http://wolfpsort.seq.cbrc.jp/) [19] To carry out protein-ligand docking, firstly three-dimensional structures of all three SAP proteins were downloaded from the AlphaFold Protein Structure Database (https://alphafold.ebi.ac.uk/) [20]. To check the quality of the 3D structures of the SAP proteins, PROCHECK was done using the SAVES server (https://saves.mbi.ucla.edu/) [21]. Using these structures, SAP proteins and Deltamethrin docking were done with the help of Autodock Vina (https://vina.scripps.edu/) [22]. Later, the obtained docked complex was further analysed using PyMol (https://pymol.org/) [23]and BIOVIA Discovery Studio v24.1.0.23298 (https://www.3ds.com/products/biovia/discovery-studio) [24].

### Insecticide exposure and SAP response

Adult susceptibility was assessed using the standard WHO tube test bioassay with diagnostic concentrations of deltamethrin (0.05%), following WHO guidelines. Healthy, laboratory-reared, 3–4-day-old, non-blood-fed adult female *An. culicifacies* were exposed to deltamethrin impregnated papers (0.05%), obtained from University Sains Malaysia, Malaysia. Negative control tests were performed using papers impregnated with silicone oil only, the solvent used for insecticide formulation. For each test, three replicates of 25 mosquitoes were exposed for 1 hour, and cumulative knock-down was recorded at 10-minute intervals. The mosquitoes were then transferred to a holding tube and fed a 5% sucrose solution. The survival test was conducted in the laboratory at the prescribed temperature (27 ± 2°C) and humidity conditions (75 ± 10%). After 1 hour of exposure, both dead and alive mosquitoes were counted, and mortality in the test and control replicates was determined to generate the survival curve using the Kaplan-Meier survival analysis method and statistical significance between survival groups was assessed using the log-rank (Mantel–Cox) test [25]. To examine molecular responses to insecticide exposure, desired tissues such as olfactory, legs, wings, and eyes were collected from the surviving mosquitoes, and *AcSAP1* expression was evaluated by using a relative quantification assay as described above, and for pairwise comparisons (Control vs. Exposed), a two-tailed unpaired Student’s t-test was applied.

### Immunofluorescence and co-localization assays

To validate the molecular expression data, we first generated polyclonal antibodies against an *in-house-*produced *AcSAP1* recombinant protein (see Supplementary Text and S4 Fig) and performed an immunofluorescence assay for cellular localization. 3–4-day-old adult female mosquitoes of *An. culicifacies* were fixed in 4% formaldehyde in PBS at room temperature for 20 minutes. For immunolabeling, the mosquito body and/or targeted tissue was permeabilized for 1 h in 0.01% Triton X-100, saturated in 1% BSA for 1 h, treated with a solution of the first antiserum overnight and then stained with a solution of the fluorescein-conjugated anti-rabbit IgG antiserum 1:80 for 1 h. All solutions were prepared in 1X PBS, and all treatments were performed at room temperature. Three washes of 5 min each with 1X PBS were included between all the treatments and at the end of the procedure [26]. The samples thus prepared were mounted on microscopic slides and observed under a confocal microscope (Olympus FLUOVIEW FV1000). The raw images of both the control and test groups were captured in identical magnifying parameters. This experiment was repeated three times with 4–5 mosquitoes as technical replicates per experiment. To illustrate and normalize the auto-florescence, the raw images were imported and processed in an isogenic graphics environment using ImageJ software, as described earlier [11].

### Statistical analysis

For statistical analysis, one-way ANOVA was applied for multi-group comparisons, followed by Dunnett’s multiple comparisons post-hoc test using the brain tissue as a control. Zeitgeber-dependent and age-dependent comparisons were tested using repeated-measures ANOVA with Dunnett’s post-hoc correction. For pairwise analyses (Control vs. Exposed), an unpaired two-tailed Student’s *t*-test was performed. Statistical significance was defined at *p* < 0.05, and exact *p*-values are reported in the figure legends. Confidence intervals (95% CI) for mean differences were also calculated. For survival analysis, mosquito survival and mortality following insecticide exposure were analyzed using the Kaplan–Meier method, and survival curves were compared using the Log-rank (Mantel–Cox) test. Hazard ratios with 95% confidence intervals (CI) were calculated to assess relative risk.

### Ethical statement

The study objective involving volunteer human participants was approved by the Institute Ethics Committee (PHB/NIMR/EC/2021/150). All adult male or female participants aged 25−50 years were provided written informed consent before participation. No individual-identifiable information is presented in the manuscript. Protocols involving animal and biosafety were also approved by the Institute Animal Ethics Committee [NIMR/IAEC/2017–1/07] and the Institute Biosafety Committee (IBSC/NIMR/2022/06), respectively.

## Results

### Age and circadian rhythm modulate SAP expression

**Fig 1:**
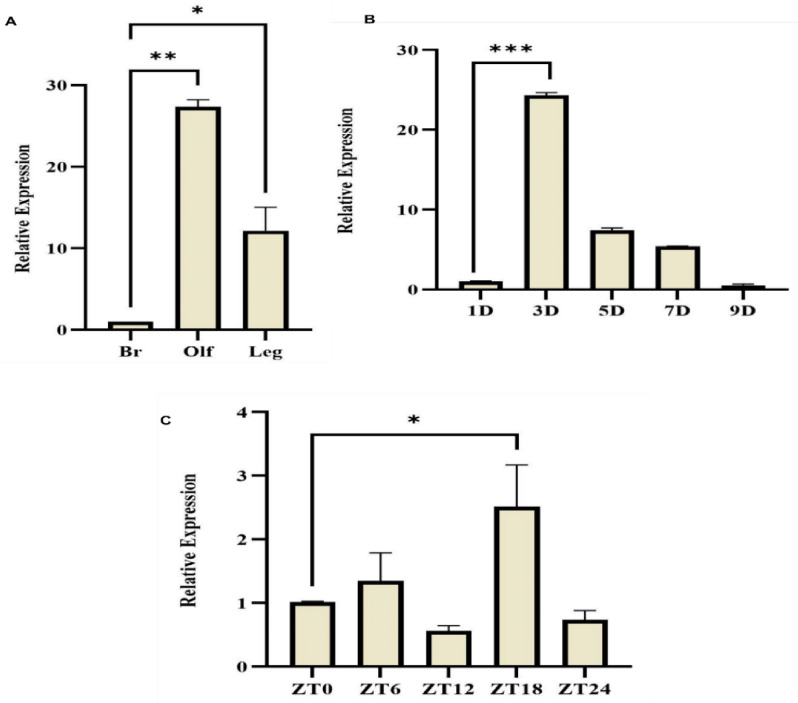
Spatial and temporal expression profiling of *AcSAP1.* (A) *AcSAP1* is dominantly expressed in the olfactory (Olf), **p < 0.001, and leg (Leg), *p < 0.0124. tissues of 3–4-day-old adult females, compared with the brain as control (n = 25, N = 3). (B) Age-dependent (D** **= days) transcriptional profiling of *AcSAP1* in the olfactory system of non-blood-fed adult female mosquitoes. Expression peaked in 3–4-day-old females and significantly reduced in 9–10-day-old females (***p < 0.0001). (C) Zeitgeber time (ZT)-dependent rhythmic expression of *AcSAP1* protein in the adult female olfactory system (OLF). Expression shows a late-night elevation in 3–4-day-old females (*p < 0.0261). Statistical significance was assessed by one-way ANOVA followed by Dunnett’s multiple comparisons test.

RNAseq analysis identified seven putative chemosensory proteins (CSPs), including three Sensory Appendages Proteins (SAP1, SAP2, and SAP3 in *An. culcifacies* (Supplementary Table 1). These proteins are low molecular weight (~12-14k Da) small peptides expressed in the olfactory tissue of the naïve 3–4-day-old adult females [8]. Expression profiling across developmental stages showed that CSPs/SAPs are expressed throughout the aquatic stages (S1 Fig). Among them, *AcSAP1* was dominantly expressed in the sensory tissues compared to other CSPs/SAPs (Fig 1A, S2 Fig). Age-dependent profiling revealed a peak expression of *AcSAP1* in the olfactory and leg tissues of 3–4-day-old females, followed by a significant reduction in 9–10-days-old females (Fig 1B). In addition, *AcSAP1* expression in 3–4-day-old females displayed late-night elevation under the Zeitgeber time scale (Fig 1C).

### *AcSAP1* influences host attraction properties

**Fig 2:**
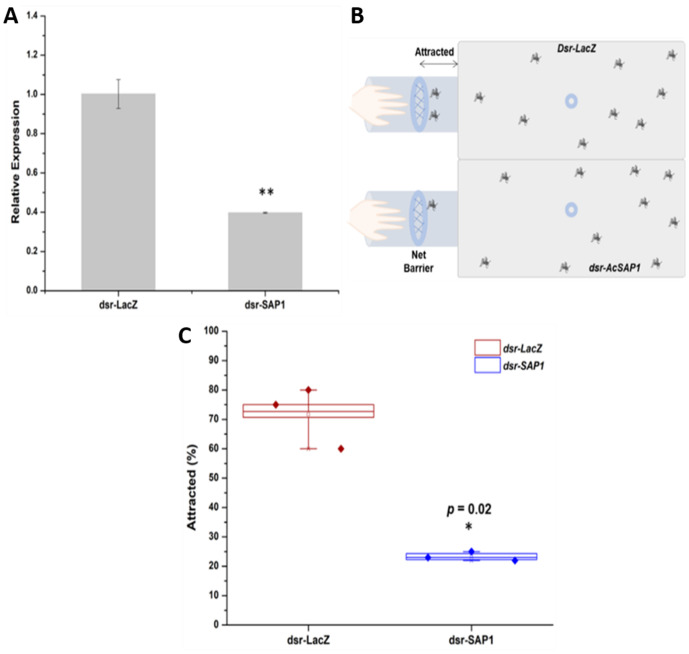
*AcSAP1* silencing interferes with host attraction properties. (A) Fold-change analysis of *AcSAP1* expression in the olfactory system of *AcSAP1*-silenced mosquitoes compared with *LacZ* dsRNA-injected controls. Knockdown efficiency was validated by qPCR, normalized against Actin, and analyzed using the 2^–ΔΔCt method. Statistical significance was assessed using Student’s t-test. (B) Schematic representation of the host-seeking efficiency assay used to evaluate the behavioral consequences of *AcSAP1* knockdown. (C) Comparative analysis of mosquito attraction to a human host in control *LacZ* dsRNA-injected versus *AcSAP1* dsRNA-injected groups. *AcSAP1*-silenced mosquitoes showed significantly reduced host attraction compared to controls (*p = 0.02, χ² test).

Given the high expression of *AcSAP1* in the sensory tissues, its role in host-seeking was examined through a behavioural assay. Control and *AcSAP1*-silenced mosquitoes were monitored for 10 minutes in host-seeking experiments (Fig 2A, B). We compared the number of mosquitoes attracted during this period. *AcSAP1-*knockdown resulted in a delayed onset of host attraction by ~2.5 minutes compared to the control group. By the end of the recording period, ~ 70 ± 10% of the control group mosquitoes were attracted to the host, while the silenced group showed a significant reduction, with 23 ± 2% fewer mosquitoes being attracted (Fig 2C). Overall, attraction efficiency in silenced mosquitoes decreased to 65 ± 6% indicating *AcSAP1’s* role in host-seeking activities.

### Insecticide exposure modulates *AcSAP1* expression

**Fig 3:**
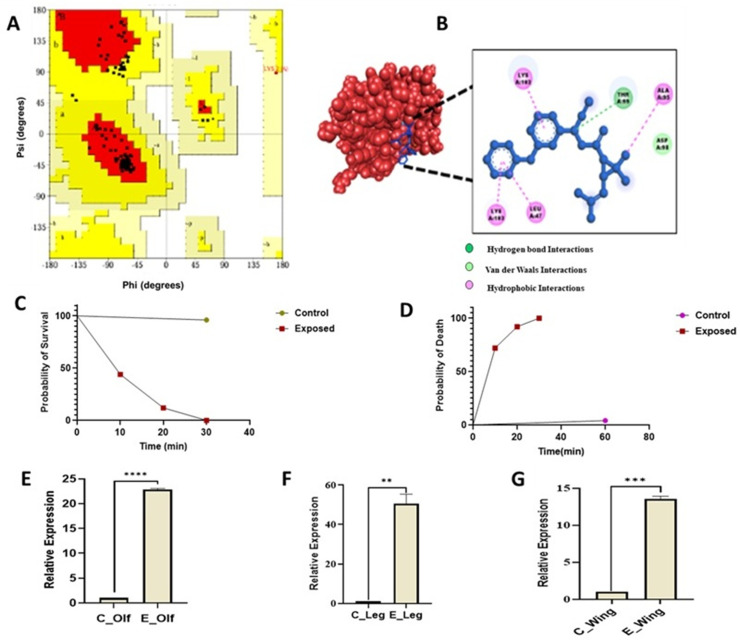
*In-silico* structural analysis of SAP-DM binding. (A) Ramachandran Plots showing SAP proteins favor DM binding as most of the atoms lie in the core regions, (also see S3 Fig). (B) Docked complexes of SAP proteins with Deltamethrin showing *AcSAP1* binding via one hydrogen bond (THR99) and four hydrophobic interactions (ALA95, LYS102, LEU47, LYS103), but showed insignificant binding to the molecule hexane used as a negative control (S3 Fig). (C-D) Kaplan–Meier survival and mortality curves highlight the survival and death profile of *An. culicifacies* adult females exposed to pyrethroid (deltamethrin, 0.05%) using the standard WHO tube test bioassay. Survival/death was recorded in a time-dependent manner, with exposed mosquitoes showing maximum survival up to 30 minutes post-exposure. Survival differences between treated and control groups were evaluated using the log-rank (Mantel–Cox) test. (E–G) Relative expression analysis of *AcSAP1* in peripheral tissues (olfactory, legs, and wings) of surviving females following deltamethrin exposure. Statistical significance was calculated using unpaired Student’s *t*-test (**p < 0.004 in legs; ***p < 0.0004 in Olf; ****p < 0.0001 in Wing). C = Control; E = Insecticide-exposed; Olf = Olfactory.

Because an insecticide-resistant strain of *An. culicifacies* is not available, we assessed the potential role of SAPs in insecticide-mediated responses using a laboratory-reared susceptible strain. *In-silico* structural prediction and molecular docking assays of SAP1, SAP2, and SAP3 with pyrethroid deltamethrin (DM) revealed that all three proteins shared conserved molecular features with some variation in their physicochemical properties (Supplementary Table 2). Ramachandran plot analysis revealed that most of the atoms were lying in the core regions, with very few in the disallowed region (Fig 3A, S3 Fig). Docking simulation indicated that all SAPs could interact with deltamethrin, with *AcSAP1* displaying the strongest binding (~5.3 kcal/mol) compared to SAP2 and SAP3 (Fig 3B, S3 Fig).

Experimental validation using the WHO tube test bioassay demonstrated that susceptible mosquitoes survived up to 30 minutes following deltamethrin exposure (Fig 3C, D). Consistent with these findings, transcriptional profiling further showed a multi-fold enrichment of *AcSAP1* expression in all the tested peripherally active tissues, including olfactory, legs, and wings, after insecticide exposure (Figs 3E–G, S4 Fig)

### *AcSAP1* mediates body sensitization against insecticide exposure

**Fig 4:**
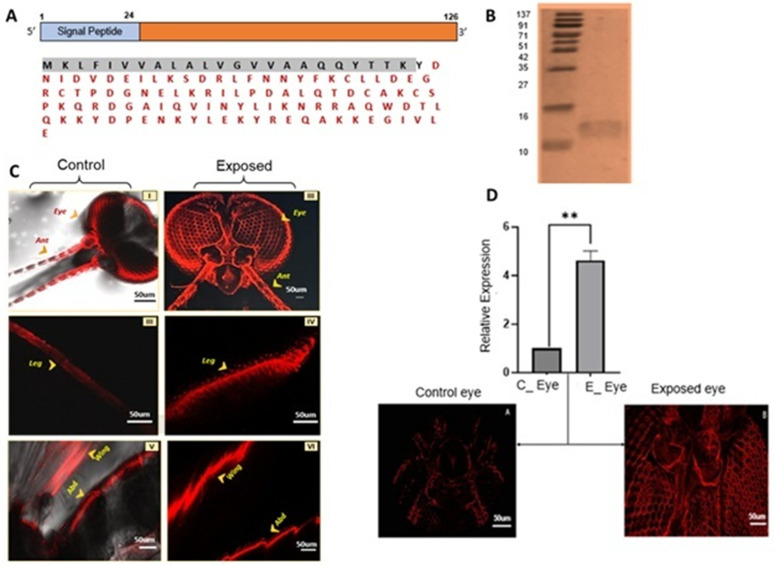
Confocal microscopy assay. (A) Schematic overview of *AcSAP1 primary* structure: Coding region with single exon shown in orange and the signal peptide in blue. The size of each region is indicated in base pairs (bp). The deduced amino acid sequence of the *AcSAP1* protein shows the predicted signal peptide (Black shaded), and the amino acids comprising OS-D in Red letters; (B) Western blot analysis of *AcSAP1* recombinant protein: Immunostaining was performed with a polyclonal antiserum against *AcSAP1*. Markers (M) size ranges from the top: 137,91,71, 51, 42, 35, 27, 16, 10kDa; (C) Comparative immunofluorescence analysis of *AcSAP1* expression in mosquito tissues under control and insecticide-exposed conditions. Representative confocal images showing the cellular localization of *AcSAP1* in adult female *An. culicifacies* tissues. Control tissues (Left panels: I, III, V) are compared with deltamethrin-exposed tissues (Right panels: II, IV, VI). *AcSAP1* was detected using fluorescently labelled secondary antibodies. Enhanced fluorescence intensity in insecticide-exposed tissues indicates an upregulation of *AcSAP1* expression in response to deltamethrin exposure. Quantitative analysis of fluorescence intensity was performed using **ImageJ software**. Background signal was subtracted, and fluorescence intensity values were normalized against the control group (see S6 Fig); (D) Relative expression analysis of *AcSAP1* in eyes to deltamethrin exposure in the adult female mosquito *An. culicifacies*. Statistical significance **p < 0.005 was calculated using Student’s t-test (n = represents the number of mosquitoes pooled for sample collection; N = number of replicates, Ant = Antennae/ Olfactory part, Abd = Abdomen sensilla).

To validate the expression of *AcSAP1* protein in the mosquito body (tissues), we first cloned, expressed, and purified recombinant protein (Fig 4A, B, S5 Fig) and subsequently generated anti-*AcSAP1* polyclonal antibodies. Immunolocalization and confocal microscopy revealed that *AcSAP1* is predominantly expressed in the sensilla of peripheral tissues. In the untreated controls, *AcSAP1* localized to specific peripheral sensilla across the body, whereas insecticide exposure resulted in markedly increased and widespread expression, including sensilla surrounding the eyes (Fig 4C, D, S6 Fig). These data indicate that insecticide exposure induces differential *AcSAP1* expression across peripheral tissues, with marked elevation around the eyes.

## Discussion

While navigating towards the host, mosquitoes rely on coordinated actions of multiple sensory systems, including olfaction, vision, and mechanoreception that operate in a sequential yet overlapping manner [27]. Host detection typically begins with long-range olfactory cues [28], followed by gradual activation of legs and wings as the mosquito approaches the host, and finally, visual engagement to identify a suitable feeding site [29].

Over the past decade, chemosensory proteins (CSPs), particularly sensory appendage proteins (SAPs), have been identified as dominant components in the olfactory and leg tissues of the malarial vectors [8,9]. In *An. culicifacies*, circadian-dependent upregulation of SAP transcripts during late-night hours supports their role in modulating host-seeking activity [8]. Our current knockdown experiments confirm this functional role, as reduced host attraction was more evident in the *AcSAP1* mRNA-depleted mosquitoes compared to the control mosquito groups. These findings align with earlier reports in *An. gambiae* that link SAP proteins to chemosensory functions and highlight their importance in vector behavioral ecology.

Insecticides are known to interfere with mosquito navigation and host-seeking pathways [30]. Our *in-silico* structural modelling and docking analysis demonstrated that *AcSAP1* exhibits strong binding affinity for the synthetic pyrethroid deltamethrin, suggesting a potential role in mediating insecticide detection and influencing mosquito behavior. This observation is consistent with earlier studies showing that odorant binding proteins can detect repellents and toxic chemicals [31–33]. Survival for up to 30 minutes of exposure provided a time window to assess the molecular and protein-level response of SAP in the *An. culicifacies* sensory tissues. Like other toxic chemical agents, insecticides also have distinct chemical properties such as bad smell, toxicity, and irritancy, which can influence both insect behaviour and human health [34]. Our data showing elevated *AcSAP1* expression in the olfactory, legs, and wings of exposed mosquitoes suggests that SAPs may participate in detecting these chemical cues. Such responses could provide an early alert mechanism to sensitize the peripherally active sensory body system/organs, thereby facilitating behavioral modification that enables *An. culicifacies* to avoid the toxic environment (Elevated SAP antibody signals in the olfactory system, legs, and wings, as well as body parts, evidenced insecticide-mediated body sensitization of the peripheral tissues. However, a striking and novel observation in our study is the pronounced induction of *AcSAP1* expression in the eyes of the pyrethroid-exposed mosquitoes. To our knowledge, such insecticide-associated ocular expression has not been reported in any mosquito species [35,36]. The increased expression around the eyes post-exposure is intriguing and suggests a potential role in mediating a response, but functional validation is needed to determine if the SAPs are active there or are simply redistributed proteins.

**Fig 5:**
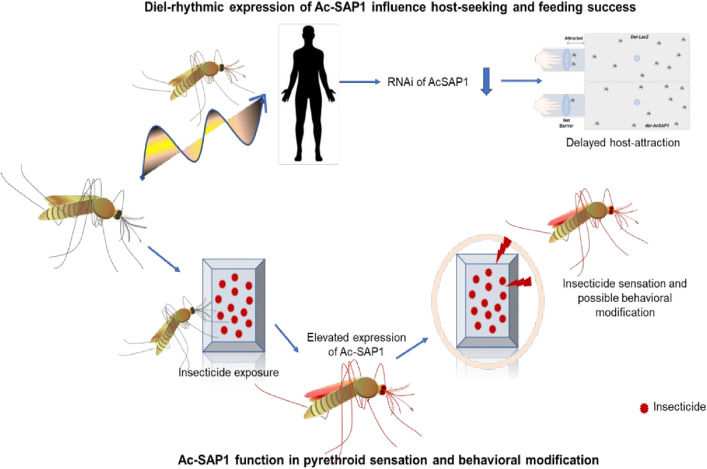
A proposed hypothesis of *AcSAP’s* role in host-seeking and behavioral modification properties. Host-seeking in *An. culicifacies* involve coordinated actions of sensory organs, including olfaction for detecting hosts, leg and wing activation for navigation, and eye engagement for locating blood-feeding sites. Sensory appendage proteins (SAPs) are crucial in this process, as evidenced by reduced host attraction in SAP-silenced mosquitoes. While our current metrics provide a good initial indication, a more thorough assessment incorporating metrics like landing attempts, probing behavior, or host choice preference could evaluate the more specific contribution of *AcSAP1*, a limitation in the current gauze barrier-based assay to host-seeking. SAPs also play a role in insecticide response, but lack direct mechanistic evidence, such as neural connectivity or signalling pathways, and warrant further research to examine their specific role in detecting and avoiding toxic environments. These findings suggest that SAPs integrate sensory information to manage navigation and insecticide avoidance, highlighting their potential as targets for innovative vector control strategies.

In summary, our findings provide novel insights into the dual functions of *AcSAP1*: (i) first evidence of circadian-regulated SAP expression, and (ii) insecticide-induced elevation across multiple sensory tissues, including the eyes, suggesting a role in early detection of toxicants in *An. culicifacies*. This aligns with nocturnal host-seeking behavior, offering fresh perspectives on behavioral modulation and its implications for malaria transmission. Our finding also builds upon prior work in *An. gambiae* [9] and other species [37], where SAPs have been implicated in insecticide resistance, by demonstrating a novel connection to circadian rhythms and sensory pre-alert mechanisms in *An. culicifacies*. A deeper understanding of SAP-mediated detection of both host and chemical cues could inform novel strategies for vector control (Fig 5). Future work should investigate the mechanistic pathways linking SAP activation to neuronal circuits and behavioral outputs, and explore whether modulation of SAP activity can enhance the effectiveness of existing interventions such as long-lasting insecticidal nets (LLINs) and indoor residual spraying (IRS).

## Supporting information

S1 File Supplementary data file. (PDF)
