# Supplementary material for: Sensory appendage protein triggers alarm to pyrethroid in Indian malarial vector Anopheles culicifacies
Source: PLoS One. 2025 Oct 7;20(10):e0333483. doi: 10.1371/journal.pone.0333483 (PMC12503281; doi:10.1371/journal.pone.0333483)

<u>Supplementary data</u>

Sensory Appendage Protein trigger alarm to pyrethroid in Indian malarial vector Anopheles culicifacies

Vaishali Saini[1,2], Pooja Rohilla[1,2], Vartika Srivastava[1,2], Gitanjali Tandon[1], Pooja Yadav[1,2], Nirmala Sankhala[1,2], Tanvi Singh[1,2], Gunjan Sharma[1,2], Suchi Tyagi[1,2], Tanwee Das De[1,3*], Rajnikant Dixit[1,2*]

S1 Table. Homology-based identified seven putative CSPs from olfactory RNAseq data of adult female mosquito *An. culicifacies*

S.N.	RNAseq Transcript-ID	Feeding Status	Label	AC-ID	Ag Homology	Ag-ID
1.	Transcript_1808	Naïve	CSP-1	ACUA010232	Chemosensory Protein (E-value: 6.00E-51) (Identity: 96.70%)	AGAP008055 (CSP-3)
2.	Transcript_447	Naïve	CSP-3	ACUA004458	Chemosensory Protein (E-value: 7.00E-71) (Identity: 96.70%)	AGAP008059 (CSP-1)
3.	Transcript_702	Naïve	SAP-3	ACUA017151 (SAP-3)	Chemosensory Protein (E-value: 1.00E-29) (Identity: 90.90%)	AGAP008054 (SAP-3)
4.	Transcript_12	Naïve	SAP-1	ACUA018714 <u>(SAP-1)</u>	Chemosensory Protein (E-value: 2.00E-40) (Identity: 99.73%)	AGAP008051 (SAP-1)
5.	Transcript_1446	30m_PBF	CSP-5	ACUA013563	Chemosensory Protein (E-value: 5.00E-80) (Identity: 99.40%)	AGAP008058 (CSP-5)
6.	Transcript_6551	Naïve	SAP-2	ACUA013409	Chemosensory Protein (E-value: 2e-76) (Identity: 98.18%)	AGAP00852 (SAP-2)
7.	Transcript_2682	30hr PBM	CSP-6	ACUA008309	Chemosensory Protein (E-value: 1.00E-66) (Identity: 99.9%)	AGAP001303 (CSP-6)

PBF= Post blood-feeding;

S2 Table. *In-silico* prediction and comparative analysis of the physio-chemical nature of SAP proteins

S. No.	Property	SAP1	SAP2	SAP3
1	Accession no. (VectorBase)	ACUA018714	ACUA013409	ACUA017151
2	Sequence length	126	127	126

**3**	Molecular weight	14617.84	14764.96	14563.70
**4**	Theoretical pI	8.29	5.34	8.57
**5**	Instability index	36.06	34.69	29.67
**6**	Aliphatic index	93.65	84.57	84.37
**7**	Grand average of hydropathicity (GRAVY)	-0.550	-0.598	-0.685
**8**	Extinction coefficients (units of M-1 cm-1, at 280 nm measured in water)	16180	16180	17670
**9**	Subcellular localization	Cytoplasmic	Cytoplasmic	Cytoplasmic

S3 Table. Highlighting representative example of detailed statistical analysis follwoed for different experimental assays.

Fig1A

Post hoc tests

Dunnett's multiple comparisons test	Mean Diff.	95.00% CI of diff.	Below threshold?	Summary	Adjusted P Value
Br vs. Olf	-26.35	-33.03 to -19.68	Yes	**	0.001
Br vs. Leg	-11.16	-17.84 to -4.491	Yes	*	0.0124

Fig1C

Dunnett's multiple comparisons test	Mean Diff.	95.00% CI of diff.	Below threshold?	Summary	Adjusted P Value
ZT0 vs. ZT6	-0.329	-1.586 to 0.9282	No	ns	0.7773
ZT0 vs. ZT12	0.4576	-0.7996 to 1.715	No	ns	0.5732
**ZT0 vs. ZT18**	**-1.494**	**-2.751 to -0.2371**	**Yes**	*****	**0.0261**
ZT0 vs. ZT24	0.2847	-0.9725 to 1.542	No	ns	0.8434

Fig:3E

Unpaired t test (Non-parametric Test)	
**P value**	**<0.0001**
**P value summary**	********
Significantly different (P < 0.05)?	Yes
One- or two-tailed P value?	Two-tailed
t, df	t=128.3, df=2
How big is the difference?	
Mean of column A	1.001
Mean of column B	22.81
Difference between means (B - A) ± SEM	21.81 ± 0.1700
**95% confidence interval**	**21.08 to 22.54**

Fig3G

**Non-parametric Test**	
Unpaired t test	
**P value**	**0.0004**
**P value summary**	*******
Significantly different (P < 0.05)?	Yes
One- or two-tailed P value?	Two-tailed
t, df	t=49.21, df=2
How big is the difference?	
Mean of column A	1.005
Mean of column B	13.56
Difference between means (B - A) ± SEM	12.55 ± 0.2550
**95% confidence interval**	**11.45 to 13.65**

**Comparison of Survival Curves**		
Log-rank (Mantel-Cox) test		
Chi square	53.59	
df	1	
P value	<0.0001	
P value summary	****	
Are the survival curves sig different?	Yes	
Gehan-Breslow-Wilcoxon test		
Chi square	45.76	
df	1	
P value	<0.0001	
P value summary	****	
Are the survival curves sig different?	Yes	
Hazard Ratio (Mantel-Haenszel)	A/B	B/A
Ratio (and its reciprocal)	0.02893	34.57
95% CI of ratio	0.01120 to 0.07469	13.39 to 89.26
Hazard Ratio (logrank)	A/B	B/A
Ratio (and its reciprocal)	0.02449	40.84
95% CI of ratio	0.01109 to 0.05407	18.50 to 90.16

S1 Fig. Developmental expression profiling of seven CSP/SAP members in the mosquito *An. culicifacies.* Relative gene expression during aquatic development, showing relative expression of identified members of CSPs/SAP transcripts in the mosquito *An. culicifacies*. The egg was considered the control for all test samples. L1-4 (larval stages) (n=10, N=3).

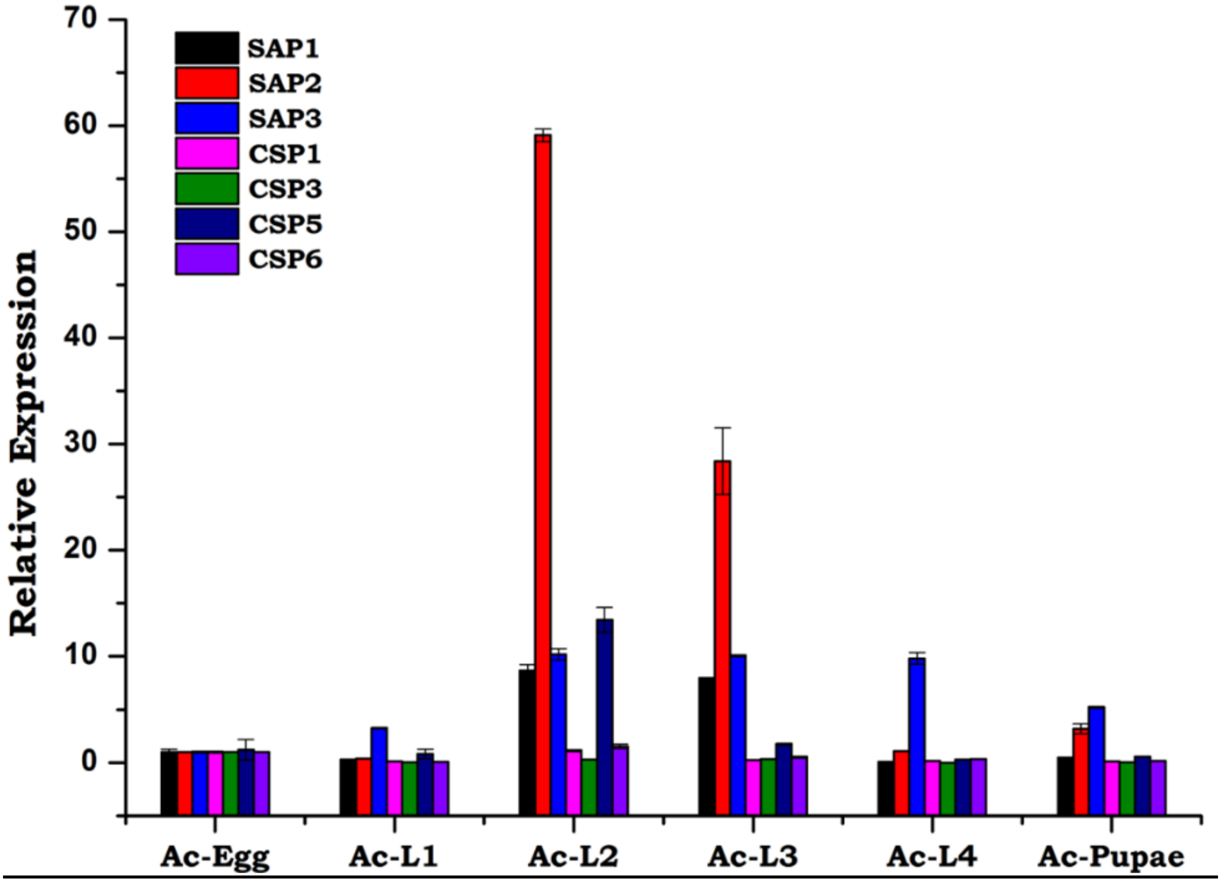

S2 Fig. **Tissue-specific Expression analysis.** CSP/SAPs differentially expressed in peripheral sensory organs: Olfactory (OLF) and Legs were dissected from 3-4 days old naïve adult female mosquitoes *An. culicifacies*. Graphs show abundant expression of SAPs in the olfactory and leg tissues. Data were compared with female brains as a control (n = 25, N=3).

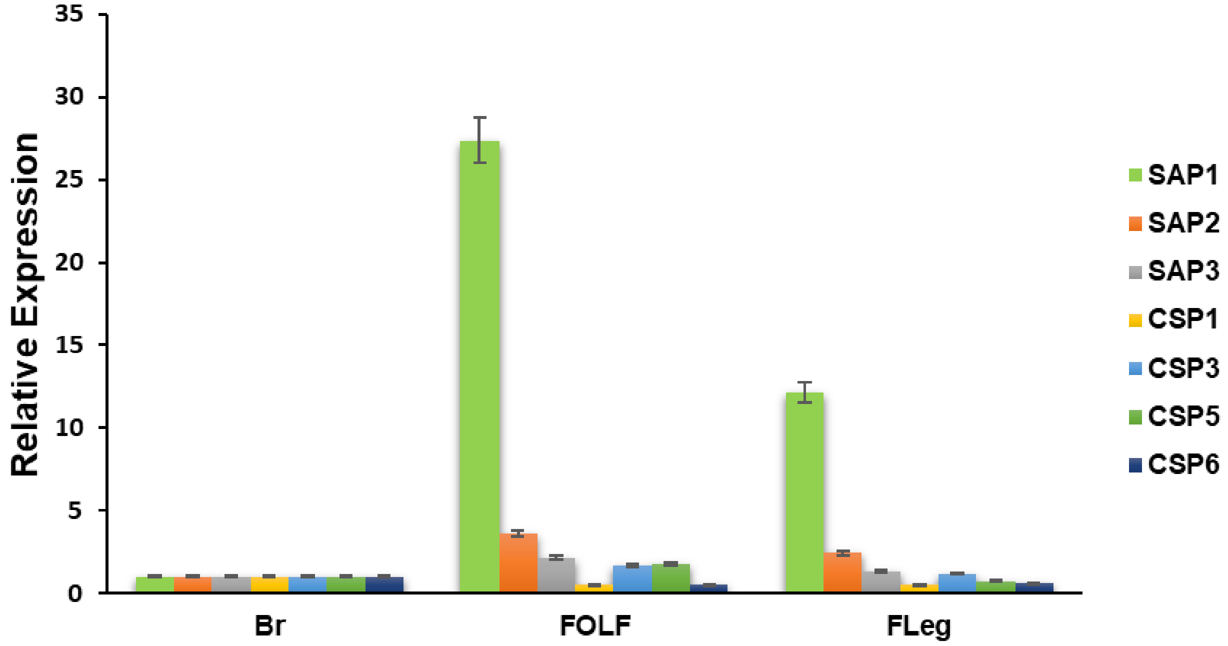

S3 Fig. ***In-silico*** **3-D structural modelling, and molecular docking analysis of SAP/insecticide/Non-insecticide interaction.** (a)Ramachandran plots for SAP2 and SAP3 proteins**.** In this figure red color indicates the core region, yellow indicates allowed regions, lemon indicates generously allowed and white is disallowed region. Docked complexes of SAP proteins with Deltamethrin: SAP2 is forming 1 hydrogen bond (TYR25) and 3 hydrophobic interactions (LEU39, PRO55) while SAP3 participating in two hydrogen bonds (ASP29, TYR43) and three hydrophobic interactions (TYR25, VAL28, PRO55). Following is the color of bond representation: Green- Hydrogen Bond, Pink- Hydrophobic interactions. (b) Interaction analysis of SAP1 with Non-insecticide ligand molecule which clearly shows two weak hydrophobic interactions between LYS44 and LEU47 residues of SAP1 with Hexane molecule

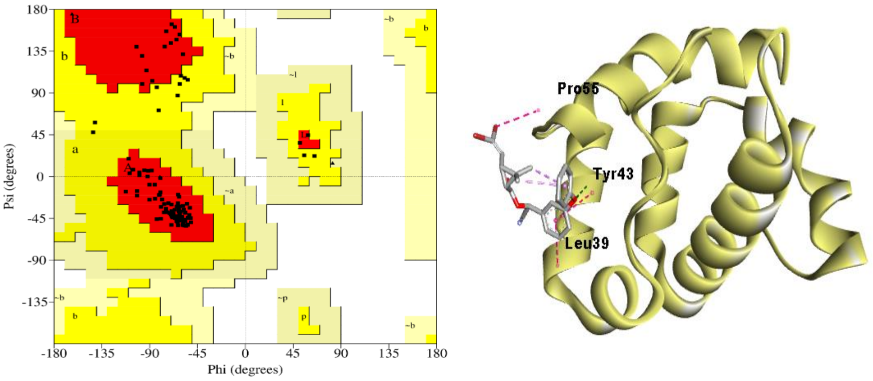

(a) SAP2 +

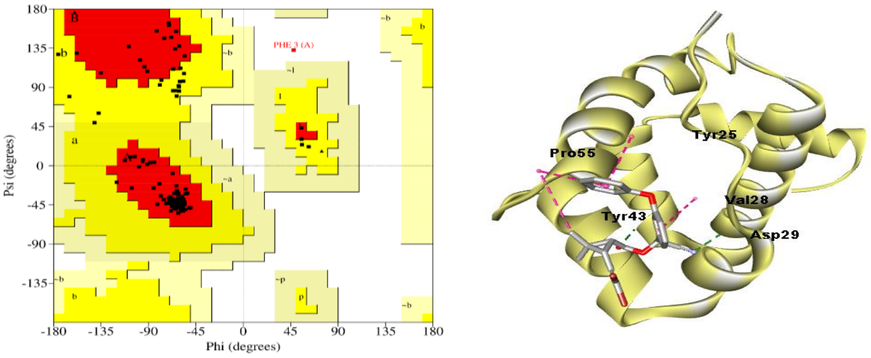

(b) SAP3 +

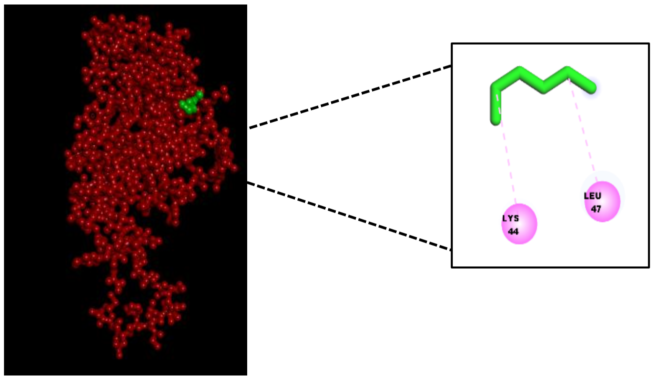

(b) SAP1 + Hexane

S4 Fig. Relative expression analysis of *AcSAP1, AcSAP2, AcSAP3* in peripheral tissues (Folf = olfactory, Fleg = legs, and Fwing =wings) of surviving females following deltamethrin exposure. C = Control; E = Insecticide-exposed; Olf = Olfactory.

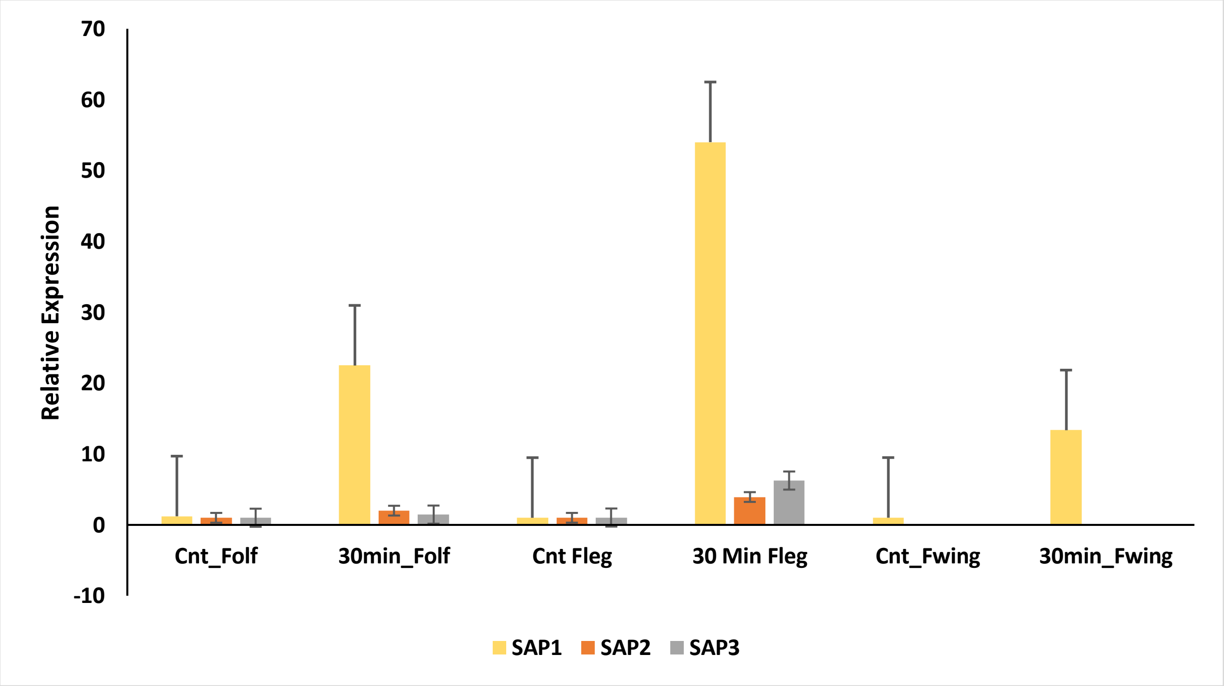

Extended supplementary Methodology Text

Cloning, bacterial expression, and protein purification: The first few amino acids were observed as signal peptides, and removed before designing cloning primers (Supplemental Fig4A). SAP1 protein was cloned into the bacterial expression vector pET28a (+) (Novagen, Madison, WI) between the Nco1-Xho1 restriction sites. The SAP1 recombinant plasmid was then extracted, verified by PCR (FigS4B)/sequencing, and then transformed into E. coli BL21competent cells. A single colony was grown overnight in 50 ml LB broth (including 50 µg/ml kanamycin). Five liters of LB medium were inoculated with the 50 ml overnight culture at 37°C for 2–3 h until the absorbance at OD 600 reached 0.6. The protein expression was then induced for 22 hours using IPTG with a final concentration of 1 mM at 18°C. The bacterial cells were harvested by centrifugation (16,000 rpm, 30 min), resuspended in a lysis buffer (buffer A - 50 mM Tris-HCl Ph 8.0, 300 mM NaCl, 10% glycerol), lysed by sonication for 20 min with 2 min interval and centrifuged again (16,000 rpm, 30 min). The supernatant was collected and mixed with equilibrated (buffer A) Ni-NTA beads and kept on a rotator for O/N. Next day O/N Ni-NTA beads packed in column and washed with 20 mM Imidazole in a buffer. Finally, the protein was eluted with 300 mM Imidazole. Collected elution fractions were pooled, concentrated up to 5-6 ml and loaded on Hi-Load 16/600 Superdex 200 column AKTA purification. Collected fractions analyzed by 15% SDS-PAGE (Fig S4C-D).

ELISA: Recombinant SAP1 (200 ng) was coated using 1X PBST buffer in 96 well microtiter plates at 4 °C overnight. Unbound protein was washed with 1X PBST and blocking was done with

2% BSA. Rabbit raised polyclonal primary antibody of different titer (1.5ng, 3.15ng, 6.25ng, 12.5ng, 25ng, 50ng, 100ng and 200ng) was incubated for 1 h at 37 °C with the coated protein. Unbound antibody was washed and incubated with HRP conjugated anti rabbit Alexa 594 secondary antibody (1:10,000). After washing, TMB detection reagent (Himedia, India) was added and the reaction was stopped using stop buffer (0.3N NH_2SO4). Absorbance was detected at 450 nm in microplate reader (Varioskan, Termo, USA).

Western blot analysis: Purified SAP1 was separated on 15% SDS-PAGE and then transferred to a PolyVinylidene Fluoride (PVDF), membrane using a machine (Genscript ALLIANZ bio). The membrane was blocked with 2% BSA in 1X TBST at room temperature and washed three times with PBST (5 min each). The blocked membrane was then incubated with the purified rabbit anti-SAP antiserum (2ug/ml) for 1 h at room temperature and washed with TBST. Subsequently, the membrane was incubated with an anti-rabbit secondary antibody (1:10K). After repeated washing, the membrane was developed with TMB and visualized with Enhanced Chemiluminescence detection reagents (GE Healthcare).

S5 Fig. (A)Deduced amino acid sequence of *AcSAP1* protein showing the predicted signal peptide (Black), the pro-peptide (pink), and the amino acids comprising OS-D in Red letters. (B) PCR and clone verification. (C) Protein induction (M -Marker, 1- uninduced, 2- whole cells, 3- Supernatant after sonication. (D) Ni-NTA purified protein was gel filtered by size exclusion chromatography HiLoad 16/600 Superdex 200 column.

A

Signal Peptide

MKLFIVVALALVGVVAAQQYTTKYDNIDVDEILKSDRLFNNYFKCLLDEGRCTPDGNELKRILPDALQTDCAK
CSPKQRDGAIQVINYLIKNRRAQWDTLQKKYDPENKYLEKYREQAKKEGIVLE

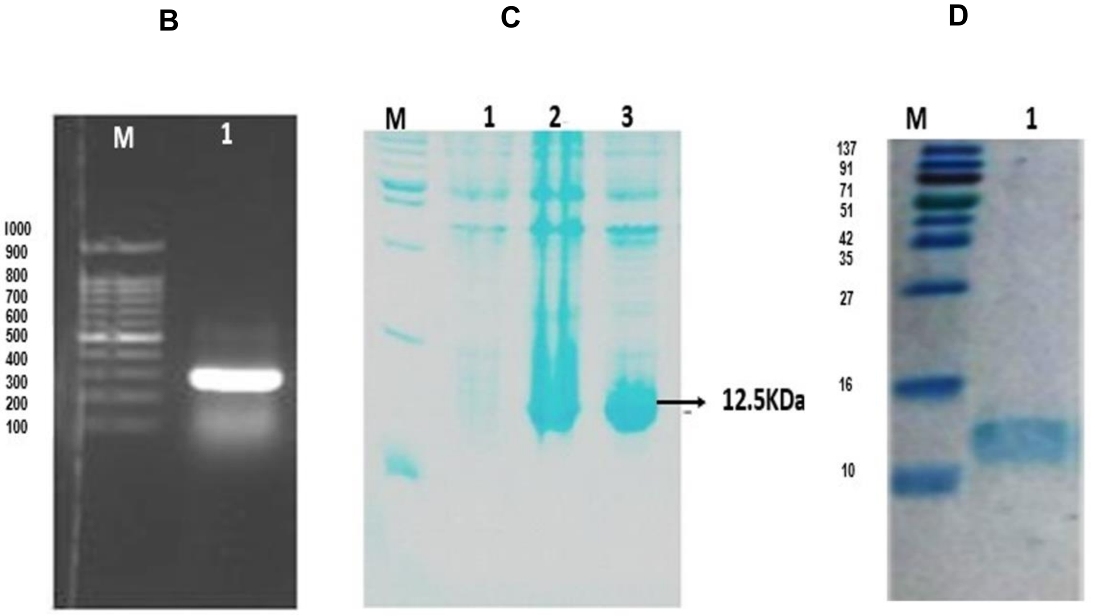

S6 Fig. Confocal images with Intensity Histogram variation. Anti-SAP1 antibody labelled images were captured under a confocal microscope, and corresponding histograms were generated to compare the fluorescent intensity variation among Control (CNT) and insecticide-exposed (EXP) mosquito's sensory tissues viz. Panel-I [Antennae (Ant) and Eyes (Eye)]; Panel-II [Wing and Abdomen (Abd)]; Panel-III [Legs]

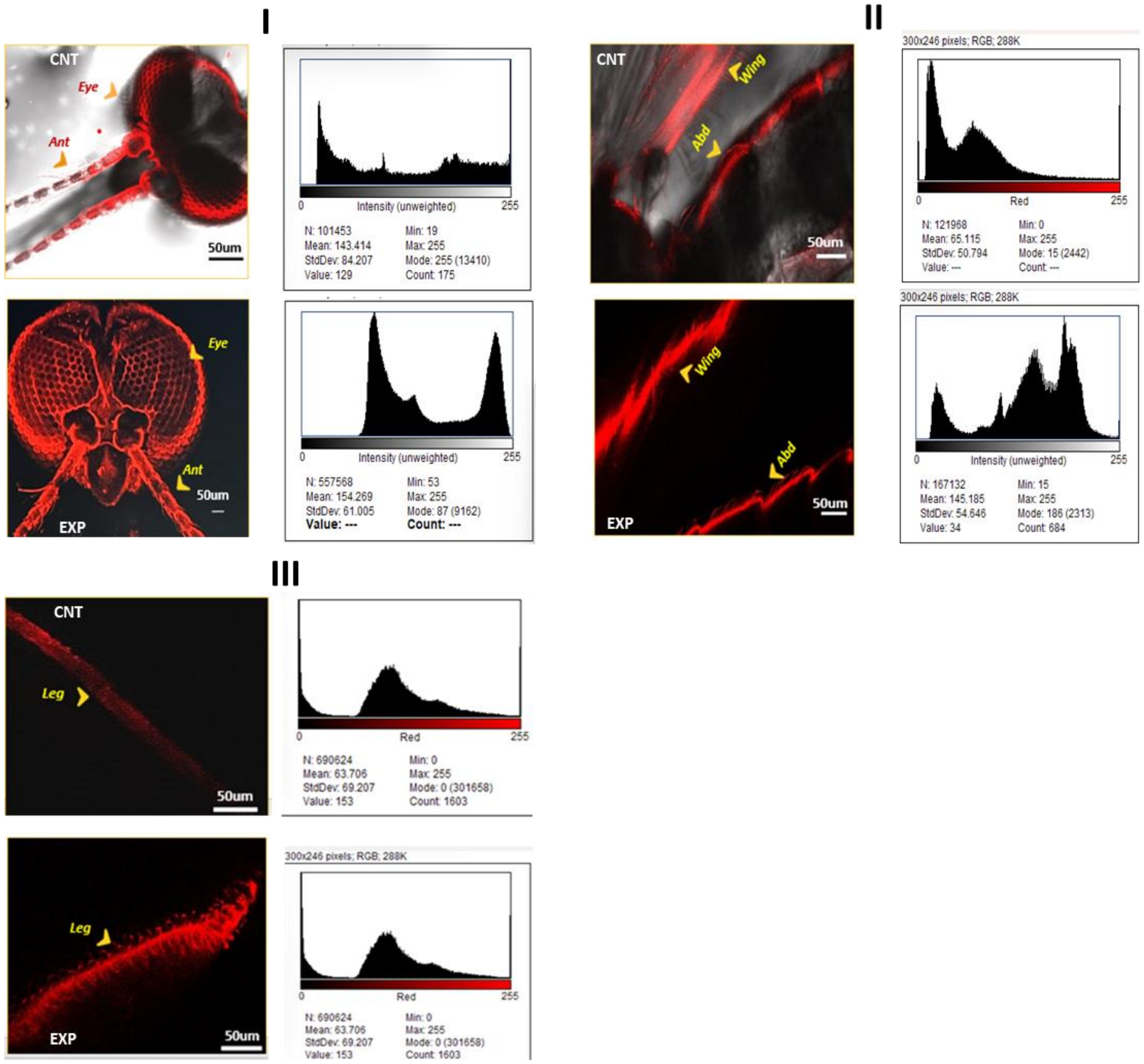

Supplement: S1 File — (PDF) [file pone.0333483.s001.pdf]
